# Dr. Jean E. Brooks

**Published:** 1913-02

**Authors:** 


Dr. Jean E. Brooks of Binghampton, Louisville, 1888, is reported deceased by the P. O. department.

